# Sacrococcygeal Teratoma

**Published:** 2013-04-01

**Authors:** Vivek Gharpure

**Affiliations:** Children's Surgical Hospital 13, Pushpanagari, Aurangabad, 431001, India

 (This section is meant for residents to check their understanding regarding a particular topic)

## Questions

1. What is the incidence of sacrococcygeal teratoma?
2. What factors are considered to modify the biologic behaviour of sacrococcygeal teratomas?
3. Discuss antenatal management of sacrococcygeal teratoma?
4. How a newborn with sacrococcygeal teratoma should be worked up?
5. Discuss surgical management of SCT.
6. Describe chemotherapy of SCT.
7. What should be the follow-up /surveillance program for SCT?
8. Discuss complications, prognosis and recurrence of SCT.
9. Discuss the management of intra-abdominal SCT.
10. How are SCT staged?
11. What is histological grading of SCT?

## Answers

1. Incidence is said to vary from 1 per 50,000 live births to 1 in 35,000 live births. But a population based study from Newcastle upon Tyne, UK has found the incidence to be 1 in 27,000 live births [1].
2. Various interdependent clinical and epidemiologic variables such as the age at diagnosis, sex, tumor site, histology which all correlate to different cytogenetic and molecular biologic aberrations; are considered to modify biologic behaviour of teratomas. Size of a sacrococcygeal teratoma (average 8 cm, range 1 to 30 cm) does not predict its biological behaviour. Histological grading of SCT does not seem to correlate with prognosis [2].
3. Antenatal diagnosis is being more commonly made nowadays. Gabra et al found that before 1988, 2 of 18 were diagnosed antenatally compared with 8 of 15 between 1988 and 2001 [1].
Fetal ultrasound and MRI are the mainstay of antenatal diagnosis of SCT. Compared with sonography, MRI more accurately characterizes the intrapelvic and abdominal extent of the tumors and provides more information on compression of adjacent organs. Additional anatomic resolution provided by MRI results in more accurate prenatal counseling and improved preoperative planning for surgical resection [1].
Tumor steals blood from fetal circulation, causing the heart to work extra hard and making cardiac failure possible. Cardiac failure manifests as hydrops, Association of fetal hydrops and SCT is usually fatal and always fatal prior to 30 weeks gestation. For the mother, there is the risk of “maternal mirror syndrome” in which the mother’s condition parallels that of the sick fetus.
Fetal hydrops (excess fluid retention from high output cardiac failure) is the sole indication for fetal surgery, the only exception being drainage/ decompression of cystic teratoma. If hydrops develops, close maternal–fetal observation is needed. If the condition of the fetus is stable with no high output cardiac failure, then only regular antenatal monitoring is required. Maternal corticosteroids are advisable to accelerate fetal pulmonary maturation. If the condition of the fetus (fetal hydrops) places him or her in jeopardy, immediate intervention is recommended. If the fetus is mature, there will be an emergency Cesarean section. Termination of pregnancy may have to be considered in cases with bad prognostic signs (cardiac failure, placentomeagaly, and marked hydrops).
In some cases fulfilling the following criteria, fetal surgery to remove the SCT will be recommended.
  - Accurate prenatal diagnosis and well defined natural history to allow the confident diagnosis of a correctable lesion that will otherwise prevent fetal survival;
  - Absence of other life threatening or debilitating anomalies;
  - Ability to perform the procedure without increased risk to the mother's life or her future fertility.
  The following factors are considered to help outcome in these cases of hydrops fetalis.
  - The increased blood volume represented by tumour circulation may have exceeded the capacity of baby's lungs to arterialise blood;
  - Venous blood from the tumour may have contributed to a low mixed venous PO2 and pulmonary vasoconstriction; excision may have removed the source of desaturated acidaemic blood and relieved pulmonary vasoconstriction;
  - Fetal ascites and abdominal distension may have embarrassed lung development in utero, resulting in reduced lung volumes;
  - Ascites raised the diaphragm after birth and paracentesis thus improved pulmonary function.
  General anesthesia is administered to provide complete pain relief for mother. Although general anesthesia will alleviate most of the pain of the fetus, additional anesthesia is provided to fetus during the operation. Uterus is partly lifted out of the abdomen. Uterus is opened and SCT is exposed. Tumor is removed and wound is closed.
  **Management at delivery**
Management of SCT depends on fetal gestational age and well being, presence of associated anomalies, tumour vascularity assessed by colour flow Doppler. Preterm labor and premature delivery may occur due to polyhydramnios (excess amniotic fluid) and/or the mass of the tumor. Highly vascular tumours should be delivered by caesarean section to avoid risk of haemorrhage during the second stage of labour. Delivery should be conducted in a tertiary hospital. Size of SCT determines mode of delivery. Non-vascular tumours and those less than 10 cm in diameter may be delivered vaginally. Tumour size has been more than 10 cm in almost all reported cases with difficult deliveries. Teratomas should be well protected after delivery because erosion of the surface may precipitate bleeding before surgical excision is undertaken. Blood loss in large and even benign tumours has been reported to be substantial, sometimes equaling the patient's blood volume. Most reviews report mortality ranging from around 5% to 9% following exsanguinating haemorrhage.
4. Baby is examined for other associated anomalies. Routine investigations are done as part of anesthesia evaluation and surgical fitness. Blood is cross matched. Abdominal ultrasound may reveal abdominal extension of tumor if the external component appears small. Additional investigations like CT scan or MRI will necessitate anesthesia and should be done only if necessary. Alpha feto protein (AFP) should be measured and interpreted carefully as normal newborns also have high levels of AFP. AFP is secreted by fetal liver and yolk sac during intrauterine life. Postnatally, neonatal liver also secretes AFP, but it rapidly falls to normal adult level (10ng/ml) by 8 month of life. In case of SCT, microscopic foci of yolk sac or embryonal carcinoma are the main source of persistently high AFP. It has prognostic significance, and persistent high levels after excision of SCT may indicate recurrence.
5. The technique of wide resection of benign lesions with coccygectomy is helpful in preventing recurrence and has changed little over the last four decades. It was concluded by Schropp et al in 1992 and stands true after 20 more years [2]. Coccyx must be excised in all patients. During surgery, precautions must be taken to prevent hypothermia, which is easily precipitated because of the large surface area and the vascularity of the tumour. Recurrence rate of 37% was reported if the coccyx was not removed completely. Patients with malignant SCT are managed after surgery with irradiation if residual disease is present, and always with combination chemotherapy. Standard surgical management includes complete excision of tumor, without rupture or spillage, without damaging bowel and bladder, with minimum resection of gluteal muscles and without injury to pelvic nerves. A chevron shaped incision is frequently used, but it could depend on the size and location of the tumor. Most tumors have a plane of dissection and can be removed without enblock excision. It is safer to catheterize the bladder to keep it away from the tumor and place a large rubber catheter in rectum for identification. Levator ani muscles are often stretched over tumor and should be reconstructed after tumor is excised. Drainage is necessary as there is a large raw area and collections should be avoided. Patient should be nursed in prone position for a week to prevent fecal soilage. Bladder can be kept catheterized till this time.
6. Wakhlu et al have stated that none of the initial 14 patients with malignant lesions survived beyond 2 years. Of the latter 11 (who received cisplatinum-based chemotherapy), 10 were alive 1 year after surgery; thus highlighting the significant improvement in survival following chemotherapy [3]. Non-fatal hematologic toxicity was common, but deafness and pulmonary and renal toxicities were rare in the UK Children’s Cancer Study Group series [4]. Standard therapy for good-risk patients is four cycles of cisplatin, etoposide plus bleomycin (BEP); cure rates are close to 90%. After chemotherapy and normalization of markers, patients should generally undergo resection of residual masses. Approximately 75% of intermediate-risk and 45% of poor-risk patients group achieve a durable complete response with BEP. Potentially curative options in the salvage setting include ifosfamide plus cisplatin-containing standard dose therapy and high-dose carboplatin plus stem-cell rescue. However, surgery remains an essential component of care [5]. For recurrent tumors, VeIP (Vinblastine, Ifosfamide, Cisplatinum) is capable of producing durable complete remissions in patients with disseminated germ cell cancer who relapse after cisplatin-etoposide-based induction therapy [6].
7. Amoah M et al concluded in his study of 18 patients from Ghana that most patients do not comply with follow-up appointments [7]. This is the most likely situation in the subcontinent too. Three monthly clinical examinations in the first year are necessary to evaluate and manage urinary and fecal incontinence, if that should develop. Rectal digital examination should be done during every visit to check for local recurrence. If alphafeto protein levels were high, they should be monitored till they reach normal and then measured at 3-6 month intervals for three years. Suspicion of recurrent disease will make further investigations like abdominal USG and CT, CXR or chest CT, tumor markers; necessary.
8. Four of 23 patients with immature teratoma and malignant foci developed recurrence, suggesting that surgical resection followed by close observation are effective treatment [8]. Calaminus et al found that in children with malignant SCT, treated with an intensive, short-interval, platinum-based regimen, the stage, extent of metastases, extension into bone, and AFP level had no prognostic significance [9]. Metastases at diagnosis and completeness of the first attempt of tumor resection were significant prognostic predictors; however, metastases were not predictive for patients treated with up-front chemotherapy [10]. Gobel et al conclude that even locally advanced and metastatic sacrococcygeal GCT can be successfully treated with up-front cisplatinum-based chemotherapy followed by delayed but complete tumor resection.
In a retrospective study of 173 patients, Derikx et al have concluded that immature and malignant histology, or incomplete resection are significant risk factors for recurrence. Mature teratoma has the biological capability to become malignant [11].
Other complications of SCT are not uncommon. Derikx et al have found that a large proportion of the patients with SCT have problems with defecation, urinary incontinence, or a cosmetically unacceptable scar that affects QoL. Patients who are at higher risk for the development of long-term sequelae cannot be clearly assessed using clinical variables [12].
Gabra et al have noted that presentation beyond the newborn period was associated with malignant disease and poorer outcome. They also found that neuropathic bladder or bowel disturbance was identified in 7 of 20 patients on long-term follow-up [13].
Rescorla et al in a study spanning 22 years and covering 126 patients found that benign teratomas have a significant recurrence rate mandating close follow-up for more than 3 years; surgical resection alone is adequate therapy for non-metastatic malignant tumors; survival for malignant lesions with metastases is excellent with modern chemotherapy [14].
The type of the teratoma and the surgical approach both contribute to functional sequelae. Tumours with a large presacral component may present with lower extremity weakness or paralysis, constipation, abdominal distension, and urinary tract symptoms secondary to bladder outlet obstruction. Isolated cases of lower extremity weakness or paralysis and bladder dysfunction have been described post-operatively, particularly in association with malignant lesions. Many of the previously reported large series did not report long term faecal and urinary incontinence. Electromanometrically detected rectal and bladder dysfunction in 40% of children following excision of SCT has been reported, though overt incontinence is rare. Tumours with large intrapelvic extensions requiring an abdomino-perineal approach for resection may be associated with a higher incidence (67%) of functional sequelae. It is difficult to determine retrospectively whether dysfunction was caused by the tumour itself or by the surgery. Others have found fecal soiling in more than 20 % patients after SCT resection. No correlation between the severity of anorectal malfunction and the degree of intra-pelvic extension of the tumour has been found. Other health problems, including urinary incontinence, were reported by 13 patients (50%).
  Hypergonadotropic hypogonadism and sperm abnormalities have been reported in men born with benign SCT. Such patients may have Leydig cell dysfunction, abnormal spermatogenesis, or both. The association of these with SCT may reflect a congenital germ cell defect.
9. Some SCT have a large abdominal component. This tumor may press on bladder, bowel and cause symptoms, compression changes in kidneys and bowel. These may also cause weakness in lower limbs. Rectal examination is done in every patient with SCT to determine deep extension. If it is not possible to reach the upper limit of tumor on rectal examination, these patients should undergo abdominal USG and or CECT to determine extent of abdominal disease. These patients often require abdominal and perineal exploration for complete resection. If possible, abdominal component is performed early, and tumor is completely mobilized. Patient is then turned prone and perineal dissection is done. Tumor can then be delivered from perineal route. Depending upon the extent of tumor and size, the approach may have to be reversed.
10. The American Academy of Pediatrics' surgical section (APPSS) classification helps in grading the extent of SCT, as follows
  - Type I—Predominantly external with minimal presacral component.
  - Type II—Present externally but with significant intrapelvic extension.
  - Type III—Apparent externally but predominantly a pelvic mass extending into the abdomen.
  - Type IV—Presacral with no external presentation.
11. SCT are graded histologically as follows
  - Grade 0—Tumour contains only mature tissue.
  - Grade 1—Tumour contains rare foci of immature tissues.
  - Grade 2—Tumour contains moderate quantities of immature tissues.
  - Grade 3—Tumour contains large quantities of immature tissue with or without malignant yolk sac elements.

## Footnotes

**Source of Support:** Nil

**Conflict of Interest:** None
